# Longitudinal evaluation of sleep disturbances in survivors of childhood cancer: a report from the Childhood Cancer Survivor Study

**DOI:** 10.1007/s11764-026-02096-0

**Published:** 2026-08-08

**Authors:** Lauren C. Daniel, Huiqi Wang, Tara M. Brinkman, Kathy Ruble, Eric S. Zhou, Oxana Palesh, Rebecca Howell, Daniel A. Mulrooney, Valerie M. Crabtree, Sogol Mostoufi-Moab, Kevin Oeffinger, Joseph Neglia, Yutaka Yasui, Gregory T. Armstrong, Kevin Krull

**Affiliations:** 1 Department of Psychology, Rutgers University Camden, 311 N 5th St., Camden, NJ 08102, USA; 2 Department of Epidemiology & Cancer Control, St. Jude Children’s Research Hospital, Memphis, TN, USA; 3 Department of Psychology and Biobehavioral Sciences, St. Jude Children’s Research Hospital, Memphis, TN, USA; 4 Department of Oncology, Johns Hopkins University, Baltimore, MD, USA; 5 Division of Sleep Medicine and Department of Psychiatry, Harvard Medical School, Boston, MA, USA; 6 Department of Pediatrics, Dana Farber Cancer Institute, Boston, MA, USA; 7 Department of Psychiatry, Virgina Commonwealth University Massey Cancer Center, Richmond, VA, USA; 8 Department of Radiation Physics, MD Anderson, Houston, TX, USA; 9 Department of Oncology, St. Jude Children’s Research Hospital, Memphis, TN, USA; 10 Division of Oncology, Children’s Hospital of Philadelphia, Philadelphia, PA, USA; 11 Department of Pediatrics, Perelman School of Medicine of the University of Pennsylvania, Philadelphia, PA, USA; 12 Department of Medicine, Duke University and Duke Cancer Institute, Durham, NC, USA; 13 Department of Pediatrics, University of Minnesota Medical School, Minneapolis, MN, USA

**Keywords:** Sleep, Childhood cancer survivors, Aging, Chronic health conditions

## Abstract

**Purpose:**

The current study examines longitudinal changes in sleep disturbances and the risks they pose for new-onset health conditions in survivors of childhood cancer.

**Methods:**

Five-year survivors (*N* = 1081; median [range] age 50.0 [45.2–55.0] years) and siblings (*N* = 214; age 49.2 [39.9–51.1] years) from the Childhood Cancer Survivor Study completed the Pittsburgh Sleep Quality Index (PSQI) at two time points (median interval = 17 years). Sex-specific changes of PSQI scores were assessed, adjusting for demographics, using generalized estimating equation. Within survivors, logistic regressions estimated associations between persistent clinically significant sleep disturbances with diagnosis and treatment exposures, and new-onset chronic health conditions.

**Results:**

Female siblings reported increases in sleep disturbances (T1 35% to T2 47%; PR = 1.30, 95% CI 1.04–1.62). However, increases among female survivors (T1 40% to T2 49%; PR = 1.25, 95% CI 0.99–1.58), male survivors (T1 34% to T2 44%; PR = 0.98, 95% CI 0.68–1.40), and male siblings (T1 24% to T2 32%; PR = 1.28, 95% CI 0.86–1.91) were not significant. Survivors reporting persistent sleep disturbances were more likely to develop new-onset hypertension (PR = 1.498, 95% CI 1.02–2.14), migraines (PR = 1.90, 95% CI 1.40–2.58), and emotional distress (PR = 8.07, 95% CI 4.07–16.00) but not subsequent malignancies (PR = 1.03, 95% CI 0.52–2.01).

**Conclusions:**

Persistent sleep disturbances are closely tied to the onset of chronic health conditions and mental health concerns.

**Implications for Cancer Survivors:**

Screening for and treating sleep disturbances early in childhood cancer survivorship has the potential to improve long-term health and mental health.

## Introduction

Currently, over 85% of children diagnosed with cancer will become 5-year survivors of their disease, such that by 2040, it is estimated that there will be more than 580,000 survivors of childhood cancer in the USA [1]. Advances in treatment and supportive care have led to this growing population of aging childhood cancer survivors [2]; yet, it is necessary to consider how age-related changes accelerated by cancer and cancer treatments may affect health outcomes. Sleep quality is closely linked to health outcomes in adults in the general population [3, 4], it can worsen with age [5], and survivors of childhood cancer report greater impairments in sleep compared to their siblings [6–9]. In the general population, it is established that the prevalence of insomnia [3] and obstructive sleep apnea [4] increase steadily with age, and these disorders are related to poor quality of life, depression [10], and cardiovascular morbidity [4], respectively. Sex differences in sleep are apparent with aging as well, with women exhibiting a greater likelihood of developing insomnia and men being more likely to develop sleep-disordered breathing conditions [11]. The association between aging and sleep is bidirectional. Aging affects sleep architecture, neurotransmitter control, and circadian rhythmicity, while disrupted sleep and/or circadian disruption can hasten aging [5].

At age 35, up to 25% of childhood cancer survivors report significant sleep disturbances following the completion of cancer treatment [6]. The frequency of insomnia and snoring in survivors is greater than that observed in sibling controls [6–9]. Among adult survivors of childhood cancer, disrupted sleep is associated with poor neurocognitive functioning [8], physical health, and anxiety in cross-sectional evaluation [9]. Longitudinally, poor sleep predicts later depressive symptoms in adolescent and young adult childhood cancer survivors but not in non-cancer peers [7], although a meta-analysis of non-cancer pediatric and young adult studies found a small effect size for sleep disturbances predicting depression [12]. Among childhood cancer survivors who are already at risk for the development of depression [13], cardiovascular conditions [14], headaches/migraines [15, 16], and secondary malignancies [17], it is important to understand if sleep disturbances are associated with physical and emotional health conditions.

The current analyses examined longitudinal changes in sleep over 17 years between cancer survivors and their siblings, stratified by biological sex. Additional analyses tested demographic and clinical risk factors for persistent sleep disturbances and associations with the development of adverse physical and mental health outcomes over time in the survivors.

## Methods

### Study population

The Childhood Cancer Survivor Study (CCSS) is a retrospectively identified and longitudinally followed cohort of 5-year survivors of childhood cancer diagnosed between 1970 and 1999 with leukemia, CNS malignancy, lymphoma, Wilms’ tumor, neuroblastoma, soft tissue sarcoma, or bone sarcoma before age 21 at one of 31 institutions in North America. A randomly selected group of siblings of survivors is also followed as a comparison group. The study was approved at each member institution by local IRBs, and participants provided informed consent to complete study measures and medical record review. A subset of the full cohort and their siblings were selected in 2002 to complete sleep measures (Time 1), with methods and the sample described previously [18]. This subset was over-representative of Hodgkin lymphoma, given the higher rate of fatigue reported in this group [19]. Sleep was again assessed in the entire CCSS cohort in 2017 (Time 2). The current analysis focuses on the survivors and siblings who completed surveys at both time points (Fig. 1).

We have previously published on the cross-sectional sleep data from 2017 (*N* = 12,340) [20], of which the current longitudinal sample represents a subset (8%). The demographic comparisons of the longitudinal and cross-sectional samples are in Supplemental Table 1. The longitudinal sample was more likely to be female (54.9% vs 51.1%), White, older, and overweight than the cross-sectional sample.

### Primary outcomes

Participants completed the *Pittsburgh Sleep Quality Index (PSQI)* at both Time 1 and Time 2. The PSQI is a 19-item self-report measure of sleep over the past month, assessing sleep duration and timing, insomnia symptoms, sleep medication use, and sleep-disordered breathing symptoms. A PSQI total score of >5 indicates clinically significant poor sleep quality [3]. Scores ranged from 0 to 21 in the current sample.

Individual PSQI items were dichotomized to indicate clinically significant sleep disturbances. *Total sleep time* was calculated and dichotomized into <6 h and ≥6 h based on evidence of poor health outcomes related to short sleep duration [21]. Clinically significant cut-points for *insomnia* were determined based on diagnostic criteria (sleep onset latency greater than 30 min, poor sleep efficiency [less than 85% of the time in bed is spent asleep], or frequent night or early morning awakenings occurring 3+ times per week) [22]. Self-report of *snoring* 3+ nights per week was used to indicate symptoms of sleep-disordered breathing [23]. Regular sleep onset of later than 1 AM indicated *delayed sleep timing*. *Sleep medication use* frequency was dichotomized by no use versus any use.

### Covariates

#### Demographic covariates

Demographic variables included sex, race/ethnicity, age at diagnosis, age at survey, and body mass index (BMI).

#### Medical covariates

Treating institution medical records were abstracted to classify the survivor’s cancer diagnosis and treatment history, including cumulative chemotherapy exposure and radiation dosimetry. Maximum radiation target dose (maxTD) was estimated for brain, chest, neck, and abdomen, as previously reported [24]. The maxTD was dichotomized for each body region, classified as moderate (<20 Gy brain or <30 Gy chest, neck, abdominal) or high exposure (≥20 Gy brain, or ≥30 Gy chest, neck, abdominal) per Children’s Oncology Group Long-Term Follow-up Guidelines [25].

Because menopause has been closely related to an increased risk of sleep disturbances [26], menstrual status in 2002 was examined as a predictor of sleep outcomes. The method for classifying menstrual status has been described previously [27]. Additionally, a subsequent diagnosis of hypertension, migraine headaches, or subsequent malignancies occurring since Time 1 (2002) were also included to evaluate the association of sleep disturbances with key medical outcomes.

#### Emotional distress

Participants completed the 18-item Brief Symptom Inventory [28] between Time 1 and Time 2 in 2014–2016. Participants responded to items on a 5-point Likert scale regarding distress over the past 7 days. *T*-scores were generated based on a population mean of 50 and a standard deviation of 10. Participants were classified as exhibiting emotional distress if the Global Severity Index was above the clinical cut-off *t*-score ≥63 or any two subscales (i.e., anxiety, depression, somatization) were ≥63.

### Statistical analysis

Continuous and categorical descriptive values of demographic factors were tabulated and compared between survivors and siblings using linear and logistic regression models, respectively, with generalized estimating equations to account for potential intra-family correlation [29].

Changes to PSQI subscale scores over time were assessed within survivors and siblings using generalized estimating equation, adjusting for sex race/ethnicity, age, and BMI. Sex-specific changes of PSQI scores over time were assessed for survivors and siblings, using generalized estimating equation, adjusting for race/ethnicity, age, and BMI. Within the survivor group, the PSQI total score was dichotomized at the clinical threshold of 5 to divide the survivors into 4 groups: (1) persistent no sleep disturbances (PSQI ≤ 5 at both time points); (2) new-onset sleep disturbances (PSQI > 5 at Time 2 only); (3) persistent sleep disturbances (PSQI > 5 at both time points); and (4) improved sleep disturbances (PSQI > 5 at Time 1 only). Two separate Poisson regression models with a robust sandwich-standard-error estimator were used to assess the associations of persistent sleep disturbances with (1) demographic and treatment exposures and (2) demographic and childhood cancer diagnosis, because treatment exposures and childhood cancer diagnosis are closely related, adjusting for sex and menopause, race/ethnicity, age at Time 2, BMI, and age at diagnosis.

New-onset chronic health conditions (hypertension, migraines, subsequent malignancies, and emotional distress) diagnosed after Time 1 and their association with the 4 patterns of sleep disturbances over time were examined using modified Poisson regression with a robust sandwich-standard-error estimator, adjusting for age, sex, and race.

All analyses were conducted using SAS version 9.4, and two-sided *p* values < 0.05 were considered statistically significant. Inverse probability weighting was applied to account for the survey design of the first sleep questionnaire and subsequent dropouts under the assumption of Missing-At-Random and weight back to the population of the CCSS original cohort.

## Results

Of those who completed Time 1 (survivors *n* = 1897; siblings *n* = 369), 56% of survivors (*n* = 1081) and 58% of siblings (*n* = 214) also completed Time 2 (Table 1). Mortality between the two time points was higher for survivors (*n* = 349, 18.4%) than siblings (*n* = 10, 2.7%). The interaction between Time 1 PSQI and group was associated with increased mortality for survivors (HR = 1.03, 95% CI 1.01–1.06, *p* = 0.014) but not siblings (HR = 1.07, 95% CI 0.84–1.35, *p* = 0.588). Excluding those who died between data collection time points, survivors who participated in Time 2 were more likely to be female (55% vs 40%, *p* < 0.001), older at Time 2 (mean age 49.9 vs 46.8, *p* < 0.001), White (92% vs 83%), older at diagnosis (mean age 11.9 vs 9.3 years, *p* < 0.001), and be Hodgkin lymphoma survivors (52% vs 38%) but less likely to be CNS tumor survivors (13% vs 24%) compared to Time 2 non-responders (Supplemental Table 2). Sibling responders were also more likely to be female (59% vs 44%) and older (mean age 49.2 vs 45.7, *p* < 0.001) than Time 2 non-responders.

Participating siblings and survivors were similar in sex, age, and BMI (Table 1). Survivors were more likely to be Black, Hispanic, and American Indian/Alaska Native than siblings. The median time between Time 1 and Time 2 surveys was 17 years (cancer survivors median 17.2, range 15.1–18.8 years; siblings median 16.6 years; 14.5–17.7 years).

### Sleep outcomes by survivor/sibling status and time

Sleep outcomes for survivors and siblings at both time points are described in Table 2. Overall, 37.4% of survivors at Time 1 and 46.5% of survivors at Time 2 had a PSQI total score above the clinical cut-off of 5. In comparison, 30.4% and 40.7% of siblings had clinically significant PSIQ scores at Time 1 and Time 2, respectively. The proportion reporting elevated PSQI scores was higher for survivors than for siblings at Time 1 (*p* = 0.051) but not at Time 2 (*p* = 0.115).

Stratifying the dichotomous PSQI total score by sex indicated that the percent of survivors reporting clinically significant sleep disturbances did not change significantly for male survivors (prevalence ratio [PR] = 0.98, 95% CI 0.68–1.40), female survivors (PR = 1.25, 95% CI 0.99–1.58), or male siblings (PR = 1.28, 95% CI 0.86–1.91; Fig. 2). However, the percentage of female siblings reporting sleep disturbances increased from Time 1 to Time 2 (PR = 1.30, 95% CI 1.04–1.62). The difference in clinically significant PSQI scores between survivors and siblings was not significant within sex (male survivors vs siblings PR = 1.37, 95% CI 1.00–1.88; female survivors vs siblings PR = 1.03, 95% CI 0.85–1.26). However, male survivors were more likely to report clinically significant PSQI scores than male siblings at Time 2 (Time 1 *p* = 0.070, T2 *p* = 0.034).

Survivors had an increased prevalence of prolonged sleep onset latency (PR = 1.40, 95% CI 1.11–1.77) and nighttime awakenings (PR = 1.39, 95% CI 1.08–1.79) from Time 1 to Time 2. Siblings exhibited an increased prevalence of clinically significant PSQI scores (PR = 1.29, 95% CI 1.05–1.58), reduced sleep efficiency (PR = 1.40, 95% CI 1.02–1.92), and increased night awakenings (PR = 1.49, 95% CI 1.08–2.04) from Time 1 to Time 2. Siblings also reported a significant increase in the prevalence of snoring from Time 1 to Time 2 (PR = 2.07, 95% CI 1.17–3.65).

### Predictors of persistent sleep disturbances

Compared to males, females with typical menstruation (PR = 1.25, 95% CI 1.10–1.42), ovarian failure with no menarche (PR = 1.61, 95% CI 1.07–2.41), premature menopause (PR = 2.11, 95% CI 1.68–2.66), or surgical menopause before Time 1 (PR = 2.02, 95% CI 1.58–2.60) exhibited an increased prevalence of persistent poor sleep (Table 3). For females taking hormones prior to Time 1, there was an increase in prevalence of persistent poor sleep compared to females not taking hormones (PR = 1.17, 95% CI 1.03–1.34).

Individuals of Hispanic ethnicity were more likely to report persistent sleep disturbances relative to non-Hispanic White survivors (PR = 1.42, 95% CI 1.17–1.73). Survivors with BMIs in the overweight (PR = 1.28, 95% CI 1.12–1.46) and obese range (PR = 1.96, 95% CI 1.73–2.22) exhibited increased prevalence rates of persistent sleep disturbances. Platinum was the only chemotherapy agent associated with an increase in persistent sleep disturbances (PR = 1.81, 95% CI 1.41–2.33), while low-dose alkylating agents (PR = 0.78, 95% CI 0.63–0.97) and vincristine (PR = 0.73, 95% CI 0.57–0.93) were associated with a reduced prevalence of sleep disturbances. Low-dose neck radiation (<30 Gy PR = 1.39, 95% CI 1.09–1.77) and high-dose abdominal radiation (PR = 1.26, 95% CI 1.02–1.55, Table 3) were also associated with increased persistent sleep disturbances. Neck radiation and chest radiation were highly collinear (correlation = 0.87); therefore, we only included neck radiation in the multivariable model.

CNS tumors (PR = 1.36, 95% CI 1.17–1.58) and soft tissue sarcomas (PR = 1.34, 95% CI 1.17–1.55) were associated with an increased prevalence of persistent sleep disturbances compared to patients with leukemia when controlling for demographic factors (Supplemental Table 3). Bone sarcoma patients exhibited a reduced prevalence of persistent sleep disturbances (PR = 0.85, 95% CI 0.72–0.99) relative to leukemia patients.

### Persistent sleep disturbances and health outcomes within survivors

One-quarter of survivors reported elevated PSQI scores at both assessment time points (persistent sleep disturbance) and 40.7% reported non-elevated PSQI scores at both time points (persistent no sleep disturbance; Table 4). Persistent poor sleepers were more likely to develop hypertension (PR = 1.48, 95% CI 1.02–2.14) and new-onset migraines over time (PR = 1.90, 95% CI 1.40–2.58). Any sleep disturbance (new, persistent, or resolved) was related to an increased prevalence of emotional distress (PR = 3.60–8.07; Table 4).

## Discussion

Almost one-quarter of childhood cancer survivors reported clinically significant persistent sleep problems over a 17-year interval. While female survivors and female siblings were similar in rates of elevated sleep problems, the prevalence of sleep disturbances for male survivors, when compared with male siblings, was higher at Time 2, suggesting that the childhood cancer experience may affect sleep in male survivors differently than male siblings. Survivors with persistent sleep disturbances were more likely to develop hypertension, migraines, and emotional distress, supporting the need for early intervention for sleep disturbances to potentially reduce adverse health conditions and improve quality of life.

A recent population-based study from Korea (*N* = 223,334) examined PSQI scores across age groups [30]. The results indicated a gradual increase in the percentage of adults with clinically significant PSQI scores with age (39% of the 30–39-year-olds, 38% of the 40–49-year-olds, 41% of the 50–59-year-olds, and 45% of the 60–69-year-olds). We observed similar gradual age-related increases, although the proportion of our survivors reporting clinically significant PSQI scores at Time 2 was higher (46%) than that of the 50-year-olds in the Korean sample and generally higher than in other population-based studies using the PSQI without stratified analyses for age, which range from 35 to 40% [31–33]. The lack of significant findings between survivors and siblings may be due to the small effect size and the smaller longitudinal sample. Our prior analyses of the full cohort [20] found that survivors were more likely to report clinically significant PSQI scores than siblings (45% of survivors vs 40% of siblings; PR = 1.20, 95% CI 1.13–1.27).

Female survivors and siblings reported poorer sleep at both time points compared to their male counterparts. Declines in sleep quality with age for women related to menopausal status have been well-documented [34], with changes to sleep beginning in the peri-menopause period and persisting for 5 years after menopause [27]. For female survivors, both surgical and nonsurgical premature menopause more than doubled the prevalence of persistent sleep disturbances. Identifying and treating sleep disturbances in the context of premature ovarian insufficiency may represent an important area for improving health and quality of life outcomes, especially given the relationship between persistent sleep disturbances and the development of chronic health conditions in this population. In our subsample of female patients taking hormone replacement therapy prior to Time 1, the prevalence of persistent sleep disturbances was increased. Without a measure of sleep prior to initiating hormone replacement, it is difficult to determine how hormone replacement influenced sleep. It is possible that women who started on hormone replacement had worse sleep to begin with, and hormone replacement therapy improved their sleep to lower but still clinically elevated levels. Further prospective study of the relationship between hormone replacement therapy and sleep in cancer survivors is warranted, as hormone replacement therapy has been found to be protective of sleep in the general population [35].

Prior research has found differences in psychological distress by sex in adult cancer survivors over time, with men experiencing more distress longitudinally than women, potentially due to men being less comfortable seeking emotional support or fewer psychosocial supports offered to men [36]. It is possible that elevated sleep disturbances in male survivors compared to siblings are an indication of ongoing elevated emotional distress related to the cancer experience. Screening for sleep concerns in male survivors may be especially important to identify survivors in need of psychosocial support for emotional distress.

BMI was a robust predictor of persistent sleep disturbances. BMI has been associated with poor sleep cross-sectionally [37] and prospectively [38], with several potential neuroendocrine mechanisms linking the two [39]. Some chemotherapy agents were associated with sleep disturbances (e.g., platinum), but the small number of participants who received each agent limits the conclusions that can be drawn.

In multivariable models, radiation exposure, specifically neck and abdominal radiation, was a significant predictor of persistent sleep disturbances. These findings differ in part from our prior cross-sectional examination of sleep in the full cohort [20]. In the cross-sectional analysis, neck radiation was associated with a reduced likelihood of snoring but was not associated with PSQI total score, whereas abdominal radiation was associated with elevated PSQI scores, snoring, and delayed sleep onset. Across both analyses, abdominal radiation was consistently associated with increased sleep-related concerns. In contrast, neck radiation was associated with reduced snoring cross-sectionally, which was hypothesized to reflect increased neck muscle patency that may have reduced the likelihood of obstruction; the longitudinal analysis demonstrated a modestly increased risk of persistent sleep disturbances. This discrepancy may reflect differences in assessed sleep outcomes or temporal patterns of sleep disturbance, and the underlying mechanism for the longitudinal association with neck radiation remains to be identified.

Patients with CNS tumors and soft tissue sarcomas had a higher prevalence of persistent sleep disturbances over time, whereas bone sarcoma patients had a lower prevalence when compared to leukemia patients. These small subsamples warrant further investigation to understand the contribution of diagnosis and treatment to long-term sleep disruption. In our larger cross-sectional analysis [20], there was no difference in PSQI total scores by diagnoses.

For survivors who persistently reported clinically significant sleep disturbances, there was an elevated prevalence of hypertension, migraines, and emotional distress, but not subsequent malignancies, suggesting an important relationship between sleep and chronic health condition development. High blood pressure has been linked to poor sleep quality in non-cancer samples [40], independent of sleep-disordered breathing in adults less than 60 years of age [41]. Poor sleep quality has also been associated with non-dipping nocturnal blood pressure, which may result in adverse cardiovascular outcomes over time [42]. A meta-analysis examining poor sleep as a risk factor for hypertension found that poor sleep quality was associated with an increased likelihood of hypertension (odds ratio of 1.48), similar to the prevalence ratio in the current study [40]. Increased sympathetic arousal, increased systemic inflammation, and hypothalamic-pituitary-adrenal axis dysregulation have all been hypothesized as mechanisms linking chronic insomnia and cardiovascular disease [43].

Associations between headaches, migraines, and sleep are less well understood. The relationship between headaches and sleep appears to be bidirectional: insomnia can trigger migraine onset and intensity, and individuals who experience migraines exhibit an increased prevalence of developing insomnia [44]. A recent study in 134 migraine patients and 70 age- and sex-matched controls found that the risk of having migraines was 3.98 times higher in poor sleepers (PSQI > 5) compared to good sleepers, higher than our current findings [45]. Despite the large body of research linking sleep and headaches, the mechanisms underlying their interrelationship are less clear, although the shared hypothalamic pathophysiology of both sleep-wake functioning and headaches likely plays a role [46].

Sleep disruption (e.g., short sleep [47] and insomnia [48]) is both an antecedent and a consequence of emotional distress [49]. In our sample, sleep and mental health were highly related with sleep disturbances conferring a 3.60–8.07 increased prevalence of mental health concerns. Determining the directionality between sleep and mental health concerns has long been a debate [49]; however, evidence from randomized trials supports treating sleep disturbances to improve symptoms of anxiety, depression, and global mental health in the general population [50]. More than one-third of survivors reported symptoms of insomnia, and these symptoms significantly increased over time. Earlier treatment of sleep disturbances has the potential to improve health and quality of life for long-term childhood cancer survivors. Cognitive behavioral therapy for insomnia (CBT-I) has been found to improve depression in adult survivors [51] and improve sleep and quality of life in childhood cancer survivors [52]. Furthermore, CBT-I has also been shown to reduce headache frequency by 6.2 days in patients with chronic migraines, compared with controls [53]. Research implementing CBT-I in medical populations is still relatively new, and the most robust trial to date did not find an impact on blood pressure [54]. Despite mixed findings, disseminating CBT-I earlier in the cancer trajectory may improve health-related quality of life outcomes for long-term survivors.

The current study details changes in sleep over approximately 17 years in a large cohort of childhood cancer survivors and their siblings. By including two time points separated by 17 years, we were able to describe high-level patterns in sleep and their relationship to health outcomes. This assessment strategy does not account for sleep or health conditions that remit between assessments. The original cohort was selected due to fatigue concerns in specific diagnostic groups (e.g., Hodgkin lymphoma) and does not represent all pediatric cancers. Thus, results may not generalize to all adult survivors of childhood cancer. Because participants in this longitudinal analysis were more likely to have risk factors associated with poor sleep (e.g., female sex, older age, overweight) compared to participants in our previous cross-sectional analysis, the prevalence estimates of sleep disturbances at T2 may be biased (i.e., overestimated). The study is also limited in the use of a single self-report measure at each time point without an objective measure. Our classification of menopausal status allowed us to estimate the impact of various levels of ovarian functioning at baseline in female survivors; however, we did not reclassify menopausal status at Time 2, which may have been more relevant to changes in sleep disturbances over time. The small number of Hispanic participants within each sex limits the evaluation of ethnicity-by-sex effects. At the same time, differential Hispanic representation between survivors and siblings is unlikely to explain the observed excess sleep disturbance among male survivors because of the small number of Hispanic participants (15 Hispanic male survivors and 1 Hispanic male sibling).

Childhood cancer survivors appear to experience sleep disturbances related to sleep quality and insomnia at earlier ages than siblings. For females, sleep concerns may be related to aging given the similar increases between groups, while for males, sleep concerns did not increase with age but appear to be higher in survivors, suggesting a potential influence of cancer history. These findings support the need for regular screening for behavioral sleep disturbances and snoring in cancer survivorship programs. Referrals to multidisciplinary sleep programs that assess and treat sleep disturbances are an important avenue to improving health outcomes in childhood cancer survivors.

## Supplementary Material

Supplemental Tables

**Supplementary Information** The online version contains supplementary material available at https://doi.org/10.1007/s11764-026-02096-0.

## Figures and Tables

**Fig. 1:**
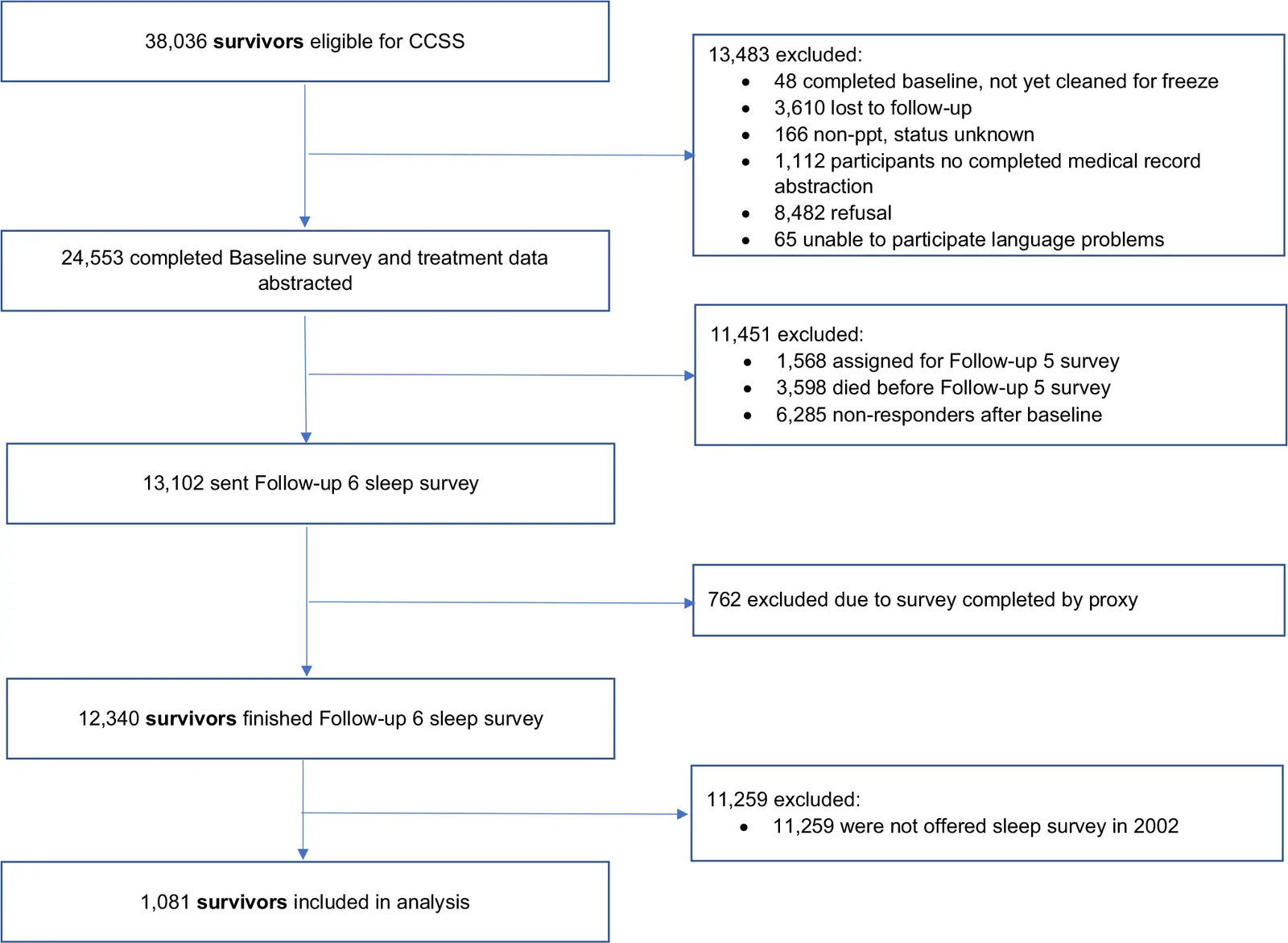

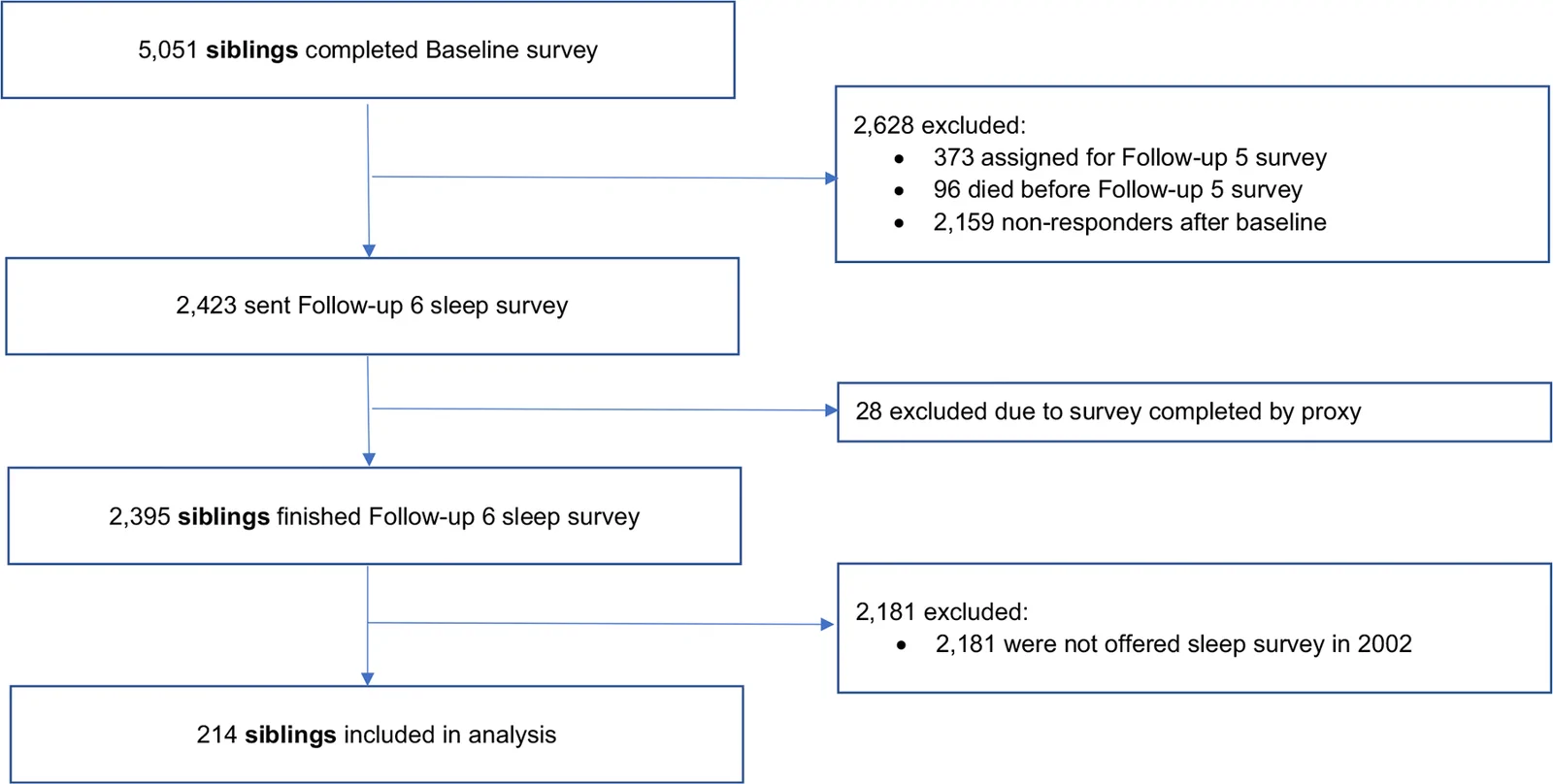
Consort Flow Diagram

**Fig. 2:**
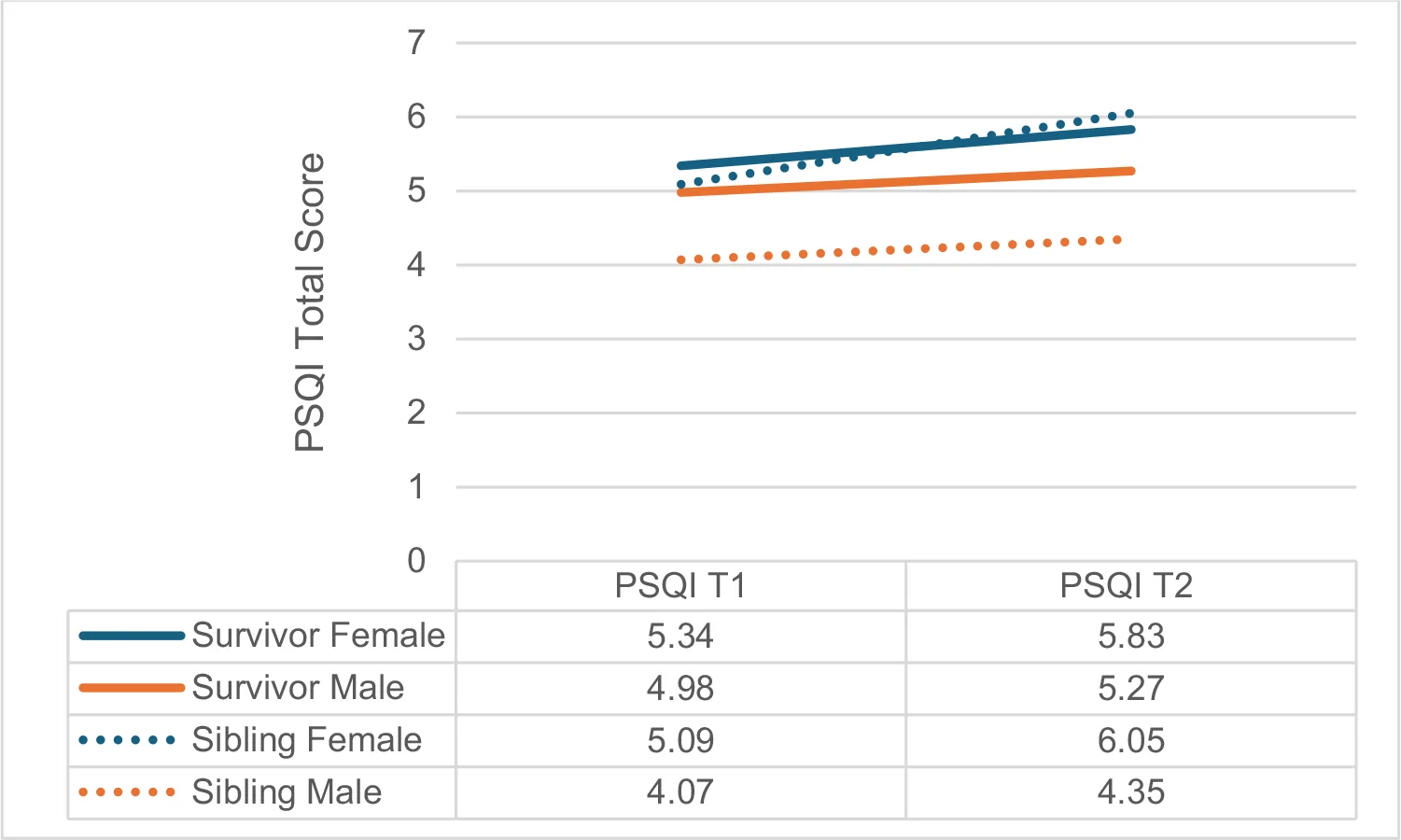
Change in PSQI total score over time by group

**Table 1:** Demographic and treatment characteristics of survivors of childhood cancer and their siblings

Characteristic	Survivors (*N* = 1081) *n* (%)	Siblings (*N* = 214) *n* (%)

Sex		
Male	487 (45.1)	88 (41.1)
Female	594 (54.9)	126 (58.9)
Race/ethnicity		
White	991 (91.7)	193 (90.2)
Black	24 (2.2)	3 (1.4)
Hispanic	39 (3.6)	4 (1.9)
Asian or Pacific Islander	9 (0.8)	3 (1.4)
American Indian/Alaska Native	4 (0.4)	0 (0.0)
Other	14 (1.3)	11 (5.1)
Age at questionnaire—Time 2		
18–29	0 (0.0)	0 (0.0)
30–39	123 (11.4)	37 (17.3)
40–49	415 (38.4)	75 (35.0)
50+	543 (50.2)	102 (47.7)
Body mass index		
Normal/underweight	431 (40.3)	82 (38.7)
Overweight	367 (34.3)	74 (34.9)
Obesity	271 (25.4)	56 (26.4)
Menopause		
All female	594 (54.9)	
Continued typical menstruation	411 (6.9)	
Acute ovarian failure	17 (2.9)	
Cranial or pituitary radiation ≥ 30 Gy	57 (9.6)	
Premature nonsurgical menopause	44 (7.4)	
Surgical premature menopause	39 (6.6)	
Age at diagnosis		
0–4	178 (16.5)	
5–9	208 (19.2)	
10–14	327 (30.2)	
15+	368 (34.0)	
Chemotherapy		
No	324 (31.8)	
Yes	695 (68.2)	
Alkylating agent (cyclophosphamide equivalent dose)		
None	463 (51.0)	
>0 to <4000 mg/m^2^	53 (5.8)	
≥4000 to <8000 mg/m^2^	95 (10.5)	
≥8000 mg/m^2^	296 (32.6)	
Anthracyclines (doxorubicin equivalent dose)		
None	702 (70.7)	
1–249 mg/m^2^	137 (13.8)	
≥250 mg/m^2^	154 (15.5)	
Vincristine		
No	434 (44.0)	
Yes	553 (56.0)	
Vinblastine		
No	872 (85.9)	
Yes	143 (14.1)	
Platinum		
No	977 (95.9)	
Yes	42 (4.1)	
Corticosteroids		
No	618 (60.8)	
Yes	399 (39.2)	
Cranial radiation		
None	811 (80.5)	
<20 Gy	39 (3.9)	
≥20 Gy	157 (15.6)	
Neck radiation		
None	484 (48.0)	
<30 Gy	156 (15.5)	
≥30 Gy	368 (36.5)	
Chest radiation		
None	504 (50.1)	
<30 Gy	142 (14.1)	
≥30 Gy	360 (35.8)	
Abdominal radiation		
None	671 (66.5)	
<30 Gy	115 (11.4)	
≥30 Gy	223 (22.1)	
New-onset chronic health condition (CHC)		
Hypertension	321 (29.7)	
Migraines/headaches	370 (34.2)	
Subsequent malignant neoplasms	108 (10.0)	
Emotional distress	124 (11.5)	

**Table 2:** Comparison of primary sleep outcomes by group and time

Sleep domain	Sleep variable	Survivor (*N* = 1081)			Sibling (*N* = 214)		
		Time 1 *n* (%)	Time 2 *n* (%)	T1 to T2 PR (95% CI)	Time 1 *n* (%)	Time 2 *n* (%)	T1 to T2 PR (95% CI)

Sleep quality	PSQI total score > 5^a,c^	404 (37.4)	503 (46.5)	1.13 (0.93–1.39)	**65 (30.4)**	**87 (40.7)**	**1.29 (1.05–1.58)**
Stratified by sex	PSQI total score > 5 – male^b,c^	164 (33.6)	214 (43.9)	0.98 (0.68–1.40)	21 (23.9)	28 (31.8)	1.28 (0.86–1.91)
	PSQI total score > 5 – female^b,c^	240 (40.4)	289 (48.6)	1.25 (0.99–1.58)	**44 (34.9)**	**59 (46.8)**	**1.30 (1.04–1.62)**
Sleep duration	Total sleep time < 6 hours^a,c^	133 (12.3)	124 (11.5)	0.67 (0.45–1.01)	21 (9.8)	22 (10.3)	0.99 (0.42–2.33)
Insomnia symptoms	Sleep onset latency > 30 min^a,c^	**314 (29.1)**	**373 (34.6)**	**1.40 (1.11–1.77)**	58 (27.1)	59 (27.7)	1.02 (0.72–1.44)
	Sleep efficiency < 85%^a,c^	291 (28.1)	373 (35.1)	1.27 (0.98–1.65)	**38 (18.3)**	**65 (31.3)**	**1.40 (1.02–1.92)**
	Night awakening/early morning awakening > 3 times per week^a,c^	**267 (25.0)**	**455 (42.8)**	**1.39 (1.08–1.79)**	**57 (26.6)**	**80 (37.9)**	**1.49 (1.08–2.04)**
Sleep-disordered breathing symptoms	Snoring > 3 times per week^a,c^	129 (12.0)	198 (18.7)	1.16 (0.77–1.76)	**19 (8.9)**	**38 (18.3)**	**2.07 (1.17–3.65)**
Delayed sleep timing	Sleep onset after 1 am^a,c^	52 (4.9)	37 (3.4)	0.94 (0.35–2.54)	10 (4.7)	4 (1.9)	0.38 (0.11–1.27)
Sleep medication use	Any medication use^a,c^	160 (14.8)	299 (28.0)	1.17 (0.85–1.62)	30 (14.0)	58 (27.4)	1.40 (0.92–2.14)

a Adjusted for age, sex, race, and BMI

b Adjusted for age, race, and BMI

c Inverse probability weighting was applied to account for the survey design of the first sleep questionnaire and subsequent dropouts under the assumption of Missing-At-Random and weight back to the participant population of the CCSS original cohort (diagnosis in 1970–1986)

Bolded values are with *p* value < 0.05

**Table 3:** Survivor demographic and clinical characteristics in association with longitudinal patterns of sleep disturbances

Variable	Sleep group status overtime				Persistent no sleep disturbances (ref) vs persistent sleep disturbances PR^a^ (95% CI)
	Persistent no sleep disturbances (*N* = 440) *n* (%)	New-onset sleep disturbances (*N* = 237) *n* (%)	Persistent sleep disturbances (*N* = 266) *n* (%)	Resolved sleep disturbances (*N* = 138) *n* (%)	

Menopause					
All male	206 (46.8)	117 (49.4)	97 (36.5)	67 (48.6)	1.00 (ref)
All female	234 (53.2)	120 (50.6)	169 (63.5)	71 (51.4)	–
Continued typical menstruation	165 (72.4)	82 (69.5)	112 (68.3)	52 (74.3)	**1.25 (1.10–1.42)**
Acute ovarian failure	5 (2.2)	3 (2.5)	7 (4.3)	2 (2.9)	1.54 (0.83–2.87)
Acute ovarian failure-no menarche	6 (2.6)	1 (0.8)	3 (1.8)	2 (2.9)	**1.61 (1.07–2.41)**
Cranial or pituitary radiation ≥ 30 Gy	24 (10.5)	13 (11.0)	12 (7.3)	8 (11.4)	1.08 (0.84–1.40)
Premature nonsurgical menopause	15 (6.6)	6 (5.1)	18 (11.0)	5 (7.1)	**2.11 (1.68–2.66)**
Surgical premature menopause	13 (5.7)	13 (11.0)	12 (7.3)	1 (1.4)	**2.02 (1.58–2.60)**
Hormone replacement before Time 1 (ref females no HRT)	90 (38.5)	48 (40.0)	73 (43.2)	30 (42.3)	**1.17 (1.03–1.34)**
Race/ethnicity					
White	407 (92.5)	213 (89.9)	246 (92.5)	125 (90.6)	1.00 (ref)
Black	11 (2.5)	5 (2.1)	4 (1.5)	4 (2.9)	0.75 (0.48–1.18)
Hispanic/Latinx	14 (3.2)	11 (4.6)	10 (3.8)	4 (2.9)	**1.42 (1.17–1.73)**
Asian or Pacific Islander	5 (1.1)	2 (0.8)	1 (0.4)	1 (0.7)	**0.22 (0.10–0.49)**
American Indian/Alaska Native	0 (0.0)	1 (0.4)	1 (0.4)	2 (1.4)	2.02 (0.47–8.79)
Other	3 (0.7)	5 (2.1)	4 (1.5)	2 (1.4)	0.76 (0.38–1.53)
Age at questionnaire					
30–39	46 (10.5)	28 (11.8)	29 (10.9)	20 (14.5)	1.00 (ref)
40–49	176 (40.0)	73 (30.8)	104 (39.1)	62 (44.9)	0.89 (0.75–1.04)
50+	218 (49.5)	136 (57.4)	133 (50.0)	56 (40.6)	0.97 (0.78–1.19)
Body mass index					
Normal/underweight	196 (45.2)	76 (32.3)	100 (38.0)	59 (43.1)	1.00 (ref)
Overweight	137 (31.6)	103 (43.8)	80 (30.4)	47 (34.3)	**1.28 (1.12–1.46)**
Obesity	101 (23.3)	56 (23.8)	83 (31.6)	31 (22.6)	**1.96 (1.73–2.22)**
Age at diagnosis					
0–4	73 (16.6)	32 (13.5)	37 (13.9)	36 (26.1)	1.00 (ref)
5–9	81 (18.4)	51 (21.5)	53 (19.9)	23 (16.7)	**1.35 (1.15–1.58)**
10–14	139 (31.6)	69 (29.1)	88 (33.1)	31 (22.5)	1.12 (0.93–1.35)
15+	147 (33.4)	85 (35.9)	88 (33.1)	48 (34.8)	1.12 (0.90–1.40)
Chemotherapy					
No	130 (31.6)	73 (32.7)	86 (34.0)	35 (26.5)	1.00 (ref)
Yes	281 (68.4)	150 (67.3)	167 (66.0)	97 (73.5)	1.12 (0.84–1.50)
Alkylating agent (cyclophosphamide equivalent dose)					
None	188 (51.2)	104 (51.5)	121 (55.5)	50 (41.7)	1.00 (ref)
>0 to <4000 mg/m^2^	21 (5.7)	14 (6.9)	11 (5.0)	7 (5.8)	**0.78 (0.63–0.97)**
≥4000 to <8000 mg/m^2^	27 (7.4)	28 (13.9)	22 (10.1)	18 (15.0)	1.09 (0.86–1.38)
≥8000 mg/m^2^	131 (35.7)	56 (27.7)	64 (29.4)	45 (37.5)	1.01 (0.87–1.18)
Anthracyclines (doxorubicin equivalent dose)					
None	282 (70.1)	156 (72.2)	174 (71.0)	90 (69.2)	1.00 (ref)
1–249 mg/m^2^	47 (11.7)	34 (15.7)	34 (13.9)	22 (16.9)	0.98 (0.83–1.16)
≥250 mg/m^2^	73 (18.2)	26 (12.0)	37 (15.1)	18 (13.8)	0.89 (0.75–1.05)
Vincristine					
No	163 (42.0)	104 (47.3)	114 (45.6)	53 (41.1)	1.00 (ref)
Yes	225 (58.0)	116 (52.7)	136 (54.4)	76 (58.9)	**0.73 (0.57–0.93)**
Vinblastine					
No	355 (87.2)	187 (83.9)	218 (86.2)	112 (84.8)	1.00 (ref)
Yes	52 (12.8)	36 (16.1)	35 (13.8)	20 (15.2)	0.88 (0.67–1.14)
Platinum					
No	397 (96.6)	212 (95.1)	242 (95.7)	126 (95.5)	1.00 (ref)
Yes	14 (3.4)	11 (4.9)	11 (4.3)	6 (4.5)	**1.81 (1.41–2.33)**
Corticosteroids					
No	248 (60.5)	136 (61.3)	154 (60.9)	80 (60.6)	1.00 (ref)
Yes	162 (39.5)	86 (38.7)	99 (39.1)	52 (39.4)	1.06 (0.90–1.24)
Cranial radiation					
None	327 (79.4)	180 (81.8)	205 (83.3)	99 (76.7)	1.00 (ref)
<20 Gy	14 (3.4)	10 (4.5)	9 (3.7)	6 (4.7)	1.08 (0.91–1.29)
≥20 Gy	71 (17.2)	30 (13.6)	32 (13.0)	24 (18.6)	0.87 (0.75–1.00)
Neck radiation					
None	201 (48.6)	103 (47.0)	108 (43.9)	72 (55.8)	1.00 (ref)
<30 Gy	66 (15.9)	28 (12.8)	45 (18.3)	17 (13.2)	**1.39 (1.09–1.77)**
≥30 Gy	147 (35.5)	88 (40.2)	93 (37.8)	40 (31.0)	0.92 (0.77–1.10)
Chest radiation					
None	207 (50.1)	104 (47.7)	118 (48.0)	75 (58.1)	
<30 Gy	61 (14.8)	27 (12.4)	40 (16.3)	14 (10.9)	
≥30 Gy	145 (35.1)	87 (39.9)	88 (35.8)	40 (31.0)	
Abdominal radiation					
None	292 (70.5)	143 (65.0)	146 (59.3)	90 (69.8)	1.00 (ref)
<30 Gy	43 (10.4)	24 (10.9)	35 (14.2)	13 (10.1)	1.17 (0.91–1.51)
≥30 Gy	79 (19.1)	53 (24.1)	65 (26.4)	26 (20.2)	**1.26 (1.02–1.55)**

a Inverse probability weighting was applied to account for the survey design of the first sleep questionnaire and subsequent dropouts under the assumption of Missing-At-Random and weight back to the participant population of the CCSS original cohort (diagnosis in 1970–1986)

Bolded values are significant at *p* < 0.05

**Table 4:** Longitudinal sleep status and associations with new-onset of specific health conditions

Group	*N* (%)	Hypertension *n* (% of 321) PR^a, b^ (95% CI)	Migraines/headaches *n* (% of 370) PR^a, b^ (95% CI)	Subsequent malignant neoplasms *n* (% of 108) PR^a, b^ (95% CI)	Emotional distress *n* (%of 124) PR^a, b^ (95% CI)

Persistent no sleep disturbance	440 (40.7)	100 (31.1) 1.00 (ref)	113 (30.5) 1.00 (ref)	41 (38.0) 1.00 (ref)	15 (12.1) 1.00 (ref)
New-onset sleep disturbance	237 (21.9)	83 (25.9) **1.46 (1.01–2.12)**	79 (21.4) 1.37 (0.94–2.01)	26 (24.1) 0.99 (0.53–1.87)	25 (20.2) **3.60 (1.67–7.75)**
Persistent sleep disturbance	266 (24.6)	99 (30.8) **1.48 (1.02–2.14)**	130 (35.1) **1.90 (1.40–2.58)**	29 (26.8) 1.03 (0.52–2.01)	63 (50.8) **8.07 (4.07–16.00)**
Resolved sleep disturbance	138 (12.8)	39 (12.2) 1.10 (0.69–1.75)	48 (13.0) 1.38 (0.91–2.09)	12 (11.1) 0.83 (0.36–1.91)	21 (16.9) **5.15 (2.27–11.68)**

a Adjusted for age, sex, and race

b Inverse probability weighting was applied to account for the survey design of the first sleep questionnaire and subsequent dropouts under the assumption of Missing-At-Random and weight back to the participant population of the CCSS original cohort (diagnosis in 1970–1986)

Bolded values are significant at *p* < 0.05

## Data Availability

The Childhood Cancer Survivor Study is a US National Cancer Institute funded resource (U24 CA55727) to promote and facilitate research among long-term survivors of cancer diagnosed during childhood and adolescence. CCSS data are publicly available on dbGaP at https://www.ncbi.nlm.nih.gov/gap/ through its accession number phs001327.v2.p1. and on the St Jude Survivorship Portal within the St. Jude Cloud at https://survivorship.stjude.cloud/. In addition, utilization of the CCSS data that leverages the expertise of CCSS Statistical and Survivorship research and resources will be considered on a case-by case basis. For this utilization, a research Application of Intent followed by an Analysis Concept Proposal must be submitted for evaluation by the CCSS Publications Committee. Users interested in utilizing this resource are encouraged to visit http://ccss.stjude.org. Full analytical data sets associated with CCSS publications since January of 2023 are also available on the St. Jude Survivorship Portal at https://viz.stjude.cloud/community/cancer-survivorship-community~4/publications.
