# Supplementary material for: Longitudinal evaluation of sleep disturbances in survivors of childhood cancer: a report from the Childhood Cancer Survivor Study
Source: J Cancer Surviv. Author manuscript; Available in PMC 2026 Oct 5. (PMC13636401; doi:10.1007/s11764-026-02096-0)
Supplement: Supplementary file 1 [file NIHMS2210528-supplement-Supplemental_Tables.docx]

**Supplemental Table 1.** Demographic comparisons of Time 2 responders and non-responders by group.

| **Variable** | **Survivors** | | | **Siblings** | | |
| --- | --- | --- | --- | --- | --- | --- |
|  | **T2 responders**  **(N=1081)**  **n(%)** | **T2 non-responders**  **(N=503)**  **n(%)** | **P-value ^a^** | **T2 responders**  **(N=214)**  **n(%)** | **T2 non-responders**  **(N=156)**  **n(%)** | **P-value ^a^** |
| Sex of patient |  |  |  |  |  |  |
| Male | 487 (45.1) | 300 (59.6) | 0.18 | 88 (41.1) | 88 (56.4) | 0.004 |
| Female | 594 (54.9) | 203 (40.4) |  | 126 (58.9) | 68 (43.6) |  |
| Race/Ethnicity |  |  |  |  |  |  |
| White | 991 (91.7) | 415 (82.5) | 0.19 | 193 (90.2) | 140 (89.7) | 0.75 |
| Black | 24 (2.2) | 25 (5.0) |  | 3 (1.4) | 5 (3.2) |  |
| Hispanic | 39 (3.6) | 33 (6.6) |  | 4 (1.9) | 3 (1.9) |  |
| Asian or Pacific Islander | 9 (0.8) | 8 (1.6) |  | 3 (1.4) | 1 (0.6) |  |
| American Indian / Alaska Native | 4 (0.4) | 7 (1.4) |  | 0 (0.0) | 1 (0.6) |  |
| Other | 14 (1.3) | 15 (3.0) |  | 11 (5.1) | 6 (3.8) |  |
| Age at Questionnaire ^b^ |  |  |  |  |  |  |
| 30-39 | 123 (11.4) | 129 (25.6) | <.001 | 0 (0.0) | 1 (0.6) | 0.002 |
| 40-49 | 415 (38.4) | 207 (41.2) |  | 37 (17.3) | 39 (25.0) |  |
| 50+ | 543 (50.2) | 167 (33.2) |  | 75 (35.0) | 70 (44.9) |  |
| Age at Questionnaire ^b^ |  |  |  | 102 (47.7) | 46 (29.5) |  |
| Mean (SD) | 49.9 (7.2) | 46.8 (7.6) | <.001 | 49.2 (8.4) | 45.7 (8.1) | <.001 |
| Median (Q1, Q3) | 50.0 (45.2,55.0) | 46.8 (39.9,52.2) |  | 49.2 (42.9,55.4) | 44.0 (39.9,51.1) |  |
| Min, Max | 33.3, 68.1 | 33.7, 67.1 |  | 32.6, 68.3 | 29.7, 63.2 |  |
| Education at baseline |  |  |  |  |  |  |
| 1-8 years (grade school) | 76 (7.3) | 65 (13.7) | <.001 |  |  |  |
| 9-12 years (high school), not graduate | 83 (8.0) | 86 (18.1) |  | 15 (7.2) | 15 (10.1) | 0.005 |
| Completed high school | 119 (11.4) | 78 (16.4) |  | 21 (10.1) | 21 (14.2) |  |
| Post high school, not college | 60 (5.8) | 22 (4.6) |  | 30 (14.5) | 24 (16.2) |  |
| Some college | 262 (25.2) | 120 (25.3) |  | 11 (5.3) | 7 (4.7) |  |
| College graduate | 329 (31.6) | 80 (16.8) |  | 41 (19.8) | 44 (29.7) |  |
| Post graduate level | 111 (10.7) | 24 (5.1) |  | 58 (28.0) | 32 (21.6) |  |
| Diagnosis |  |  |  |  |  |  |
| Leukemia | 174 (16.1) | 118 (23.5) | <.001 |  |  |  |
| CNS | 136 (12.6) | 120 (23.9) |  |  |  |  |
| HD | 563 (52.1) | 190 (37.8) |  |  |  |  |
| Soft tissue sarcoma | 92 (8.5) | 42 (8.3) |  |  |  |  |
| Bone cancer | 116 (10.7) | 33 (6.6) |  |  |  |  |
| Age at Diagnosis |  |  |  |  |  |  |
| 0-4 | 178 (16.5) | 150 (29.8) | <.001 |  |  |  |
| 5-9 | 208 (19.2) | 146 (29.0) |  |  |  |  |
| 10-14 | 327 (30.2) | 99 (19.7) |  |  |  |  |
| 15+ | 368 (34.0) | 108 (21.5) |  |  |  |  |
| Age at Diagnosis |  |  |  |  |  |  |
| Mean (SD) | 11.9 (5.6) | 9.3 (5.7) | <.001 |  |  |  |
| Median (Q1, Q3) | 12.9 (7.2,16.2) | 8.2 (4.4,14.5) |  |  |  |  |
| Min, Max | 0.0, 21.0 | 0.0, 20.8 |  |  |  |  |
| Time since diagnosis |  |  |  |  |  |  |
| Mean (SD) | 38.0 (4.6) | 37.5 (4.6) | 0.99 |  |  |  |
| Median (Q1, Q3) | 37.3 (34.1,41.5) | 36.8 (33.4,41.2) |  |  |  |  |
| Min, Max | 30.9, 48.4 | 31.0, 48.4 |  |  |  |  |
| ^a^ : Inverse probability weighting was applied to account for the survey design of the first sleep questionnaire and subsequent dropouts under the assumption of Missing-At-Random and weight back to the participant population of the CCSS original cohort (diagnosis in 1970-86).  ^b^: Age at questionnaire was predicted based on date of birth and median date of completion of FU 6 for FU6-nonresponders | | | | | | |

|  | **Survivors** | | | **Siblings** | | |
| --- | --- | --- | --- | --- | --- | --- |
| **Characteristic** | **Longitudinal**  **(N=1,081)**  **n (%)** | **Cross-Sectional**  **FU6 only**  **(N=11,259)**  **N (%)** | **P-value** | **Longitudinal**  **(N=214)**  **n (%)** | **Cross-Sectional**  **FU6 only**  **(N=2,181)**  **N (%)** | **P-value** |
| Sex |  |  | 0.0162 |  |  | 0.5858 |
| Male | 487 (45.1) | 5503 (48.9) |  | 88 (41.1) | 939 (43.1) |  |
| Female | 594 (54.9) | 5756 (51.1) |  | 126 (58.9) | 1242 (56.9) |  |
| Race/Ethnicity |  |  | <.0001 |  |  | 0.5174 |
| White | 991 (91.7) | 9448 (83.9) |  | 193 (90.2) | 1941 (89.0) |  |
| Black | 24 (2.2) | 574 (5.1) |  | 3 (1.4) | 48 (2.2) |  |
| Hispanic | 39 (3.6) | 842 (7.5) |  | 4 (1.9) | 78 (3.6) |  |
| Asian or Pacific Islander | 9 (0.8) | 180 (1.6) |  | 3 (1.4) | 20 (0.9) |  |
| American Indian / Alaska Native | 4 (0.4) | 41 (0.4) |  | 0 (0.0) | 8 (0.4) |  |
| Other | 14 (1.3) | 174 (1.5) |  | 11 (5.1) | 86 (3.9) |  |
| Age at Questionnaire-Time 2 |  |  | <.0001 |  |  | <.0001 |
| 18-29 | 0 (0.0) | 2243 (19.9) |  | 0 (0.0) | 219 (10.0) |  |
| 30-39 | 123 (11.4) | 4426 (39.3) |  | 37 (17.3) | 582 (26.7) |  |
| 40-49 | 415 (38.4) | 3229 (28.7) |  | 75 (35.0) | 699 (32.1) |  |
| 50+ | 543 (50.2) | 1361 (12.1) |  | 102 (47.7) | 681 (31.2) |  |
| Body Mass Index |  |  | 0.0475 |  |  | 0.6384 |
| Normal/underweight | 431 (40.3) | 4793 (43.4) |  | 82 (38.7) | 850 (39.5) |  |
| Overweight | 367 (34.3) | 3404 (30.8) |  | 74 (34.9) | 687 (31.9) |  |
| Obesity | 271 (25.4) | 2848 (25.8) |  | 56 (26.4) | 616 (28.6) |  |

**Supplemental Table 2.** Comparison of cross-sectional cohort and longitudinal cohort.

**Supplemental Table 3.** Multivariable model for persistent sleep disturbances with cancer diagnosis group.

| **Variable** | **Category** | **Persistent No Poor Sleep (ref)**  **vs Persistent Sleep Problem**  **PR^a^ (95% CI)** |
| --- | --- | --- |
| Sex and Menopause | Male | 1.00 (ref) |
|  | Female-AOF | **1.79 (1.05 - 3.08)** |
|  | Female-AOF-no menarche | **1.67 (1.16 - 2.41)** |
|  | Female-Cranial or pituitary radiation >= 30Gy | 0.82 (0.65 - 1.04) |
|  | Female-Normal | **1.14 (1.04 - 1.26)** |
|  | Female-PM | **2.18 (1.81 - 2.64)** |
|  | Female-Surgical PM | **1.98 (1.62 - 2.43)** |
| Race/Ethnicity | White | 1.00 (ref) |
|  | Black | **0.44 (0.29 - 0.67)** |
|  | Hispanic | **1.21 (1.02 - 1.44)** |
|  | Asian or Pacific Islander | 0.19 (0.09 - 0.42) |
|  | American Indian / Alaska Native | 1.62 (0.39 - 6.67) |
|  | Other | 1.49 (0.90 - 2.45) |
| Age at Questionnaire | 18-29 |  |
|  | 30-39 | 1.00 (ref) |
|  | 40-49 | **0.76 (0.66 - 0.87)** |
|  | 50+ | 0.87 (0.73 - 1.03) |
| Body Mass Index | Normal/underweight | 1.00 (ref) |
|  | Overweight | **1.40 (1.25 - 1.56)** |
|  | Obesity | **1.87 (1.68 - 2.09)** |
| Age at Diagnosis | 0-4 | 1.00 (ref) |
|  | 5-9 | **1.19 (1.04 - 1.37)** |
|  | 10-14 | **1.20 (1.02 - 1.41)** |
|  | 15+ | **1.39 (1.16 - 1.67)** |
| Diagnosis | Leukemia | 1.00 (ref) |
|  | CNS Tumor | **1.36 (1.17 - 1.58)** |
|  | Hodgkin’s disease | 1.04 (0.91 - 1.19) |
|  | Non-Hodgkin lymphoma |  |
|  | Kidney (Wilms) |  |
|  | Neuroblastoma |  |
|  | Soft tissue sarcoma | **1.34 (1.17 - 1.55)** |
|  | Bone cancer | **0.85 (0.72 - 0.99)** |

^a^: Inverse probability weighting was applied to account for the survey design of the first sleep questionnaire and subsequent dropouts under the assumption of Missing-At-Random and weight back to the participant population of the CCSS original cohort (diagnosis in 1970-86).

**Bolded values** are significant at p<.05.
